# Targeting Angiogenesis With Peptide Vaccines

**DOI:** 10.3389/fimmu.2019.01924

**Published:** 2019-08-08

**Authors:** Michal A. Rahat

**Affiliations:** ^1^Immunotherapy Laboratory, Carmel Medical Center, Haifa, Israel; ^2^The Ruth and Bruce Rappaport Faculty of Medicine, Technion-Israel Institute of Technology, Haifa, Israel

**Keywords:** angiogenesis, peptide vaccines, adjuvant, cancer, VEGF, EMMPRIN

## Abstract

Most cancer peptide vaccinations tested so far are capable of eliciting a strong immune response, but demonstrate poor clinical benefits. Since peptide vaccination is safe and well-tolerated, and several indications suggest that it has clear potential advantages over other modalities of treatment, it is important to investigate the reasons for these clinical failures. In this review, the current state of the art in targeting angiogenic proteins via peptide vaccines is presented, and the underlying reasons for both the successes and the failures are analyzed. The review highlights a number of areas critical for future success, including choice of target antigens, types of peptides used, delivery methods and use of proper adjuvants, and suggests ways to achieve better clinical results in the future.

## Introduction—Why Target Angiogenesis?

The unprecedented success of checkpoint inhibitors in the therapy of solid tumors has ignited renewed interest in immunotherapy as a strategy to eradicate tumor cells and prevent metastasis. However, even recent interventions such as the checkpoint inhibitors anti-CTLA4 and anti-PD-1 that “release the brakes” and mobilize effector T cells into the tumors so they can eradicate tumor cells, show limited clinical success, with only about 20–40% of the patients responding to checkpoint inhibitors as monotherapy (1). Patients treated with monoclonal antibodies that attack tumor antigens and patients treated with tyrosine kinase inhibitors (TKIs) that inhibit receptor signaling pathways, are still experiencing tumor recurrence and progression, and suffer from high mortality rates (2). Therefore, the search for an adjuvant therapy that improves survival rates is expanding, with targets other than tumor cell proteins being considered.

Tumor cells depend on angiogenesis, the process of generating new capillaries from pre-existing blood vessels, to supply them with oxygen and nutrients, remove waste products, and support tumor survival, progression, invasion and metastasis. Therefore, it has been suggested to target proteins that mediate this process. In normal physiological conditions, angiogenesis occurs during development, menstrual cycle, or wound healing, and depends on the balance between pro- and anti-angiogenic factors. However, when this balance is disrupted and pro-angiogenic factors begin to accumulate, an “angiogenic switch” occurs to initiate pathological angiogenesis, associated with many types of chronic inflammatory diseases, including cancer (3). This causes the activation, proliferation and migration of endothelial cells (ECs) through the basement membrane and extracellular matrix (ECM), using matrix metalloproteinases (MMPs) to degrade them. The migrated ECs then spatially reorganize to form tube-like structures that may mature into functional vessels. In cancer, these vessels are typically leaky due to increased permeability and lack of sufficient stabilization and maturation via attachment of pericytes (3), and are usually more complex, dilated, tortuous, and in a state of chronic inflammation (3, 4).

Targeting angiogenesis and EC mobility as an anti-tumor strategy, as was first suggested by Judah Folkman (5), may offer additional benefits. First, ECs play an important role in establishing the immunosuppressive tumor microenvironment (TME). The heterogeneous vessel density produces irregular blood flow that generates hypoxia in some regions, which is the driving force of the angiogenic switch and tumor cell metabolism via activation of the transcription factor hypoxia-induced factor-α (HIF-1α) (3, 6–9). Thus, targeting ECs may indirectly affect tumor metabolism. Second, the chronic production of angiogenic factors suppresses adhesion molecules (e.g., ICAM-1, E-selectin, CD34), thus making the infiltration and adhesion of T cells into the tumor more difficult, increasing immune suppression (10, 11). Third, tumor ECs may also actively assist in the killing of Fas-expressing effector T cells, but not T regulatory cells (Tregs), by expressing Fas ligand (FasL) (12). Fourth, tumor cells utilize several strategies to escape immune recognition, including the alteration or loss of MHC/HLA class I molecules, leading to the inability of CD8^+^ cytotoxic T cells (CTLs) to attack them (13). As ECs are genetically stable, they express class I molecules, present angiogenic targets, and allow CTLs to attack them thus causing vasculature damage (14). Thus, attacking the tumor vasculature indirectly leads to tumor cell death, as the latter are deprived of their oxygen and nutrients.

The most potent pro-angiogenic factor is vascular endothelial growth factor (VEGF), and VEGF itself or its signaling pathway have been targeted by monoclonal antibodies or their fragments (e.g., bevacizumab/Avastin, Ranibizumab/Lucentis), soluble receptors (e.g., ziv-aflibercep/Zaltrap, ramucirumab) and small molecules receptor TKIs (e.g., sorafenib, sunitinib, and others). However, these agents were proven insufficient, as their effect was transitory and moderate, they exhibited off-target toxicities and reduced delivery of chemotherapeutic agents (15–17). More importantly, upon withdrawal of treatment tumors demonstrated a more aggressive phenotype of enhanced growth, invasion and metastasis, known as the “rebound effect” (18, 19), probably because of compensatory pathways activated by other VEGF family members, pro-angiogenic factors and cytokines (4, 20). Alternatively, other, more immediate mechanisms may compensate for reduced angiogenesis, such as vessel cooption, vessel intussusception, or vasculogenic mimicry to sustain tumor blood flow and bypass the effect of the angiogenesis inhibitors (4, 20). Thus, a different approach to the targeting of angiogenesis that yields long-lasting effects is needed.

Several vaccination strategies and delivery systems have already been tried, including recombinant proteins, fusion proteins, DNA vaccines, pulsed dendritic cells and whole endothelial cell vaccines (11). However, in this review I focus only on the progress made in peptide vaccination that elicits an immune response against angiogenic targets, and I do not discuss other forms of vaccination or other mechanisms of action used by peptides (e.g., inhibition, competition), which have already been addressed by other reviews (20–23).

## Principles of Peptide Vaccination

Tumor cells are in constant interaction with immune cells, especially macrophages, as explained by the concept of immunoediting (24). The contributions of immune cells to the killing of tumors in early stages, to the shaping of the tumor during the equilibrium stage, and to the support of tumor growth in later stages [by secreting immunosuppressive and pro-angiogenic factors to the tumor microenvironment (TME)], suggests an intricate relationship between the cell types. However, although suppressed, the potential to recognize and eliminate tumor cells inherently exists even in late stages of tumor escape, as suggested by the presence of autoantibodies found in many cancer patients (25), and by the release of pre-existing CTLs from immune suppression by checkpoint inhibitors (26). Thus, the goal of any form of immunotherapy is to restore the ability of adaptive immune effector cells to attack and eradicate the tumor.

Most current immunotherapeutic approaches rely on passive immunization by introducing monoclonal antibodies directed against tumor antigens or against checkpoint co-inhibitory molecules. The advantages of monoclonal antibodies are their high specificity and affinity to tumor antigens, thus avoiding off-target toxicity. However, antibodies are very costly to develop and to produce, and they must be provided in repeated injections of high doses. In addition to the rebound effect known to arise especially in antibodies and TKIs directed against angiogenic targets (15), antibodies might also lose their effectiveness over time, as anti-drug antibody (ADA) response develops against the antigen-binding site of the therapeutic antibody and confers resistance to treatment (27, 28).

In contrast, peptide vaccination depends on active vaccination that elicits a strong immune response with memory, which may be critical to prevent tumor recurrence. This strategy is generally considered a simpler approach, with high specificity, reduced costs, easy synthesis, which is safe and well-tolerated, as detailed below. Nonetheless, despite their ability to elicit a strong immune response, cancer peptide vaccines have so far yielded only limited clinical benefits. This is mostly explained by central and peripheral tolerance mechanisms, which limit the T cell repertoire able to recognize self-antigens only to low-affinity T cells, and by the immunosuppressive TME (29, 30). Additional strategies that the tumor may employ to escape immune recognition, such as reduced MHC class I expression (30, 31) or loss of IFNAR expression (32), may also take a part in this general failure to use cancer peptide vaccination effectively.

### Choice of Antigens

The choice of the target antigen is critical to the success of the vaccination. Ideally, the target should be highly expressed only on tumor cells, to ensure that even low-affinity effector T and B cells could recognize it and mount an effective immune response. Tumor antigens are classified as tumor-specific antigens (TSAs) and tumor-associated antigens (TAAs). Viral antigens are a class of TSAs unique to tumors that originate from viral transformation, such as in the case of HPV or EBV. However, most tumors arise due to genetic instability, and different mutations (translocations, frame-shift mutations or point mutations) may generate a new protein, a truncated protein, or expose a previously hidden (crypt) epitope that are different from the normal self-protein. Therefore, such mutations that generate neoantigens could potentially be recognized by the immune system. A correlation was found between high tumor mutation load and anti-tumoral response, positive clinical response, survival, and response to checkpoint inhibitors therapy, which strengthens this premise (33). The idea to elicit an immune response against neoantigens is a promising “personalized medicine” approach, but its clinical translation may be challenging and require a multistep process (22). This process includes mapping the tumor exome, assessing the immunogenicity of specific mutations *in silico*, selecting peptide(s) that are predicted to match to the patients HLA class I and II molecules, synthesizing the selected peptide(s) under Good Manufacturing Practice (GMP) conditions and injecting them to the patient (34). Thus, tailoring the vaccine to each patient may increase the response rates, but this approach is time consuming, labor-intensive and still costly.

In contrast to TSAs, TAAs are self-antigens that are aberrantly overexpressed on cancer cells but physiologically expressed on some normal cells. For example, cancer-testis (CT) antigens are expressed on male gametes, silenced in normal adult tissues and reactivated in tumor cells (e.g., MAGE-A, NY-ESO-1, and SSX-2). Differentiation antigens are specific to a cell lineage or a tissue (e.g., Melan-A/MART-1, gp100, tyrosinase). If overexpressed in the tumor, these antigens may become immunogenic when their expression exceeds the threshold required for TCR recognition and CD4^+^ T helper activation. Antibodies directed against TAAs found in the serum of cancer patients suggest that such recognition occurs, even without treatment (25). Most peptide vaccination approaches to date were designed to target TAAs such as the CT antigen 1B (CTAG1B), MAGE family member 3 (MAGE-3), TTK protein kinase (TTK), Wilms tumor 1 (WT1), survivin (BIRC5), EGFR, erb2/Her2, indoleamine 2,3-dioxygenase (IDO1), and others (30). However, such antigens are not necessarily critical for tumor survival, and may be suppressed when attacked.

Another approach would be that of targeting proteins that regulate angiogenesis or regulate the interactions between stroma and tumor cells that promote pathological angiogenesis. This may provide universal targets to indirectly attack tumor cells, especially in combination with other treatment modalities, and reduce vaccination costs (22).

### Types of Peptides Used for Vaccination

Two types of peptides are typically used for peptide vaccination. Short peptides (<15 amino acids long, usually 9–10 amino acids) have a short half-life and are rapidly degraded in the serum. These peptides can be loaded onto the HLA class I groove from the outside of the nucleated cell, even without prior processing in professional antigen presenting cells (APCs). This may lead to tolerance or to a short-term induction of CD8^+^ T cells, without parallel induction of CD4^+^ T cells and without induction of memory (35, 36). Therefore, they are often conjugated to a carrier protein, to allow uptake and processing by APCs and to elicit an effective immune response. In contrast, synthetic long peptides (SLPs) (>20 amino acids), are more stable and immunogenic. Since they are efficiently taken-up and processed by dendritic cells (DCs), they can present the epitope in the context of both class I and class II molecules, resulting in a strong, long-lasting, and balanced anti-tumoral immune response that involves CD4^+^ T cells, CD8^+^ T cells and antibody production by B cells (35, 36). Some studies use the multiple epitope approach, where several epitope peptides are mixed and injected together, or where a single long peptide that contains several epitopes is injected. Targeting several epitopes derived from different antigens simultaneously may circumvent the ability of the tumor cell to evade immune recognition by losing an antigen.

The magnitude and strength of the immune response depends on the interaction of the peptide with the presenting MHC/HLA molecule, and even small changes in the peptide sequence may affect this interaction. Therefore, several modifications of the peptide sequence were investigated. Some studies substituted a single amino acid in the peptide sequence to better anchor the peptide to the MHC groove and enhance the T cell response (35, 37). Other modified peptides, called mimotopes or altered peptide ligands (APL), mimic the spatial structure of the presented epitope, and not necessarily its sequence. However, although mimotopes/APL elicited a better expansion of T cells than the unchanged peptide, these T cells did not efficiently cross-react with the native antigen or presented a reduced affinity relative to the native epitope (37), requiring additional boost vaccination with the native tumor antigen to improve anti-tumor immunity (38). Yet, although this approach increased the *ex vivo* CD8^+^ T cells responses in melanoma patients, it did not extend the patients' overall survival (39). This could be due to the limited number of MHC-peptide complexes exhibited by tumor cells and the lack of expression of co-stimulatory molecules (37). Multiple antigenic peptide (MAP) represents a different approach to peptide modification. Here, the peptide epitope is conjugated four or eight times onto a core of lysine residues, generating a branched peptide tree with a molecular weight of a small protein (40). This structure endows the peptide with high stability (41, 42) and increases its immunogenicity due to the increased concentrations of the repeated peptide sequence and the changes in the three-dimensional structure (43).

### Peptide Delivery and the Role of Adjuvants

Peptides can be delivered by direct subcutaneous injections in the presence of an adjuvant, or by re-infusing DCs that have first been isolated from peripheral blood, matured and expanded *ex vivo*, and then pulsed with the peptide. Both approaches yield comparable results in terms of eliciting immune responses and clinical responses (44). Novel delivery systems that were used to enhance the efficiency of vaccination include liposomes, virus-like particles that do not include the viral genome, and caged proteins nanoparticles that are self-assembled protein structures, that can be delivered with or without adjuvants (45). The different forms of nanoparticles show improved uptake by DCs, and may enhance antigen presentation on these cells (46).

Adjuvants are necessary to protect peptides from fast degradation, to prolong the release of the peptide (the depot effect) and therefore the duration of the immune response, and to recruit and stimulate APCs to process and present the peptides to B and T cells (47). The most used adjuvant for human subjects in pathogen vaccination are aluminum salts (Alum), but as these promote Th2 responses, they are not compatible with cancer vaccines (47). Thus, in human cancer patients, the most used adjuvant is Incomplete Freund's adjuvant (IFA) or Montanide ISA-51, water-in-oil emulsions of the antigen that form a depot that slowly releases the antigen. However, in some cases, the slow release of short peptide vaccines promotes secretion of pro-inflammatory cytokines (e.g., IFNγ), which in turn, enhance Fas ligand (FasL) expression on tissue cells and T cell apoptosis, exhaustion and reduced memory formation (48). This leads to persistence of T cells in the vaccination site, inhibiting their movement to the tumor, and therefore, sufficient anti-tumoral responses are not mediated (36, 49). Thus, the commonly used adjuvants could be contributing to the limited clinical success observed with peptide vaccination so far, despite the presence of peptide-specific CD8^+^ T cells in the circulation.

Alternative adjuvants that could first recruit leukocytes to the vaccination site, support T cell expansion and activation, and promote their migration to the lymph nodes and tumor site, could potentially include bacterial or synthetic TLR ligands, cytokines and growth factors, or nanoparticles that deliver the antigen (50, 51). Currently, TLR ligands, such as unmethylated CpG motifs that activate TLR9, or polyI:C that binds to TLR3, show improved immune responses to peptide vaccinations alone or with Montanide ISA-51, increased Th1 polarization and CTL responses (51). Inclusion in the adjuvant formulation of cytokines, such as GM-CSF that enhances DCs and macrophage proliferation, or IL-12 that enhances IFNγ production in T cells and NK cells, improved immune responses to peptide vaccination (51, 52). However, in most cases GM-CSF provided only weak adjuvant properties (47), and since it can potentially expand the MDSCs population and increase immunosuppression in high doses, it is recommended to use it in in low and repeated doses (51, 52).

## Angiogenic Targets for Peptide Vaccination

As mentioned before, most cancer peptide vaccines used so far were directed against TAAs, and only a few angiogenic proteins were targeted. In contrast to neoantigens, these targets are shared between many types of tumors, they are not subject to genetic variations, and they are expressed on stroma cells, such as ECs. VEGF and its receptors stand out as the main targets of this class of proteins, but other potential targets were also tested, especially in preclinical studies (summarized in Table 1).

**Table 1:** Preclinical trials for pro-angiogenic peptide vaccines.

**Antigen**	**Type of peptide**	**Cancer type**	**Adjuvant used**	**Timing**	**Result**	**References**
VEGF	Long peptide (79 aa), two cysteine residues replace with alanine	Melanoma	Raffinose fatty acid sulfate ester (RFASE)	Prophylactic^a^ vaccination	Inhibition of tumor growth by ~50%	(10)
VEGF	MAP2-dRK6	Colorectal	None	Therapeutic vaccination	Inhibition of tumor growth by ~65%	(53)
VEGFR2	Epitope screening of 38 short peptides (9–10 aa each)	A2/K2 transgenic mice implanted with different mouse tumor cells	IFA	Therapeutic vaccination	Inhibition of tumor growth by ~5-fold	(14)
VEGFR1	Epitope screening of 40 short peptides (9–10 aa each)	A2/K2 transgenic mice implanted with different mouse tumor cells	IFA	Therapeutic vaccination	Inhibition of tumor growth by ~2-fold	(54)
Fibronectin, ED-A fragment	Recombinant fusion peptide (<100 aa) conjugated to bacterial thioredoxin	metastatic mammary adenocarcinoma (MMTV-PyMT)	Montanide ISA-720 with GpC oligo	Therapeutic vaccination	Inhibition of tumor growth by ~40%	(55)
Heparanase	Octa-branched MAP with a 15 aa peptide	Hepatocarcinoma (HCC-97H cells)	CFA/IFA	Therapeutic passive vaccination	Reduced tumor volume by ~3-fold; Reduced pulmonary metastasis by 10-fold	(56, 57)
FGF-2	Heparin binding domain of FGF-2 (44 aa peptide)	B16BL6 and experimental lung metastasis models	Liposomes and lipid A	Prophylactic vaccination	Inhibition of 96% of macroscopic metastases	(58)
EMMPRIN/CD147	Octa-branched MAP with a 9 aa peptide	A498 renal, CT26 colon and TRAMP-C2 prostate carcinomas	CFA/IFA	Prophylactic and Therapeutic vaccination	Inhibition of tumor growth by 72% and 94%.	(59)

a *Prophylactic, vaccination was carried out before injection of tumor cells; Therapeutic, vaccination was carried out after injection of tumor cells*.

### Pre-clinical Studies

VEGF itself or its receptors can be obvious angiogenic targets. In a pre-clinical study, Wentick et al. (10) used a 79 amino acid long peptide that includes critical areas in the VEGF molecule, including the typical cysteine-knot fold. This sequence reconstitutes the complete conformation of the discontinuous binding site of bevacizumab to VEGF_165_. Furthermore, to prevent oxidative folding of the peptide, two cysteine residues were substituted for alanine, thus modifying the peptide. Vaccination with this peptide produced antibodies that were cross-reactive with VEGF and comparable to bevacizumab, and inhibited tumor growth in two mouse models (10). A similar approach included engineering a conformational shorter peptide of 23 amino acids that correctly mimics the VEGF binding site to VEGFR2 and includes an insertion of two cysteine residues to allow cyclization of the peptide. This peptide (VEGF-P3-CYC) inhibited the proliferation, migration and tube formation, as well as VEGFR2 phosphorylation in human umbilical vein endothelial cell (HUVEC) *in vitro*, and when injected to the transgenic VEGF^+/−^Neu2-5^+/−^ mouse model, the peptide significantly delayed tumor growth (60). In a follow-up study, the authors vaccinated mice with a Her2 peptide, and after tumor implantation, VEGF mimic peptides (the VEGF-P3-CYC and the same sequence synthesized in reverse with D-amino acids-RI-VEGF-P4-CYC) were weakly intravenously injected to the tumor-bearing mice. The combination of these two peptides resulted in a marked inhibition of tumor growth relative to each single treatment or to the controls, as well as inhibition of cell proliferation and reduction of microvascular density in the tumors (61). However, in both studies, the VEGF mimic peptides were injected to the tail vein in PBS and in the absence of adjuvant. Thus, only their anti-angiogenic functions, including inhibition of proliferation, VE-cadherin expression and angiogenesis in an aortic ring assay were measured, whereas the ability to elicit an immune response with both humoral and cellular responses was not checked.

Another approach to target VEGF was demonstrated by developing a VEGF mimotope that was identified by using a phage display technology followed by screening the library with bevacizumab/Avastin (62). Although the peptide sequence was not identical to VEGF, it mimicked the spatial organization of the epitope, suggesting that Avastin recognizes a discontinuous conformational epitope on VEGF. The resulting 12 amino acid peptide was conjugated to KLA and used to immunize mice who developed high titer of VEGF-specific antibodies that blocked VEGF binding to VEGFR2. The purified Ab inhibited the proliferation of HUVEC cells, their ability to migrate and to form tubes (62).

Kim et al. report on inhibition of VEGF using an antagonizing branched dimeric peptide with two repetitions of the six amino acids peptide sequence RRKRRR (RK6) synthesized as D-amino acids (MAP2-dRK6). This modified MAP peptide had increased stability to serum proteolysis and inhibited the binding of VEGF to its receptors expressed on HUVECs, more than the L-amino acid counterpart or the unmodified linear peptides. MAP2-dRK6 inhibited VEGF-induced, but not bFGF-induced, proliferation and signaling in HUVECs, as well as their ability to form tube-like structures *in vitro*. In a model of SW480 human colorectal cancer cells implanted in nude mice, MAP2-dRK6 could inhibit tumor growth by 65%, and reduce the microvessel density, suggesting that it effectively blocked angiogenesis (53). However, as MAP2-dRK6 was injected *s.c*. daily for 14 days without the presence of adjuvant, and tumors were monitored for only 20 days, this study did not examine a possible activation of the immune system.

In an attempt to identify the best peptide sequences to target in human VEGFR2 or VEGFR1, a library of peptides of 9–10 aa long was synthesized according to their predicted binding affinities to HLA-A0201 or HLA-A2402. Peptide-specific cytotoxic T cells (CTLs) were identified by their ability to kill HLA-restricted target cells that were pulsed with each peptide. Leading peptides for each receptor matched to the specific HLA molecule were also identified by the ability to drive cytotoxicity and IFNγ production of peptide-pulsed target cells incubated with spleen cells derived from peptide-vaccinated A2/k2 transgenic mice that express HLA-A0201 (14, 54). Lastly, the selected HLA-A0201-restricted peptides were used to vaccinate A2/Kb transgenic mice implanted with several tumor cell lines (that do not express HLA and are therefore not targeted themselves) to demonstrate *in vivo* efficacy. Inhibition of tumor growth suggests that targeting angiogenesis *in vivo* could be a feasible strategy (14, 54). These experiments helped identify the VEGFR1-1084 and VEGFR2-169 peptides that were subsequently used in clinical trials.

FGF-2 (or bFGF) is a potent pro-angiogenic factor that promotes ECs proliferation by binding either to the FGF receptor or to heparin sulfate proteoglycan on the cell surface. Vaccinating mice with an FGF-2-derived peptide (44 aa long) directed to the heparin binding site domain, but not with a peptide (22 aa long) directed to the receptor binding site domain, administered in liposomes containing lipid A, resulted in generation of high titer of FGF-2 specific antibodies. Moreover, the heparin domain peptide inhibited neovascularization in an angiogenesis sponge model, and reduced metastatic foci by 96% in the lungs of vaccinated mice (58).

Fibronectin (FN) is a complex ECM protein that has many isoforms due to alternative splicing. Interestingly, the specific FN type III extracellular domains A and B (ED-A, ED-B) are only expressed during vasculogenesis in the embryo and are spliced out in adult normal tissue. However, they are expressed again in high levels in tumors, especially near angiogenic vasculature (55). Here, Femel et al. therapeutically vaccinated the transgenic MMTV-PyMT mice model of metastatic mammary adenocarcinoma with a construct consisting of the ED-A fragment (<90 aa) conjugated to bacterial thioredoxin (TRX). They demonstrate a significant 40% reduction in primary tumor weight and reduction in metastases relative to control mice, with increased infiltration of macrophages into the tumors. While CD31-stained blood vessels were not reduced in number, their functionality was compromised by the vaccination, as more fibrinogen leaked out of the vessels and less FITC-labeled lectin was perfused (55). Surprisingly, although the titer of anti-ED-A antibodies was significantly elevated, the authors do not mention any attempt to examine a CD8^+^ T cell response as well.

Heparanase is the only endoglycosidase found that specifically degrades and removes heparan sulfate (HS) side chains from heparan sulfate proteoglycans, thus releasing heparin-binding proteins to the TME. It is expressed by tumor- and activated stroma-cells including ECs, activated only in acidic conditions that are typical to the tumor TME, and in addition to regulating ECM remodeling it has a role in activating signaling pathways that increase transcription of pro-angiogenic factors, such as VEGF (63, 64). Testing of the passive vaccination against heparanase was reported in two papers, where rabbits were immunized with a 15-amino acids sequence derived from human heparanase that was synthesized as octa-branched MAP. The resulting polyclonal antibodies were then purified from rabbit serum and injected to mice bearing the HCC-97H hepatocarcinoma tumor in different doses. The antibodies reduced the serum levels of VEGF and FGF and decreased MVD, tumor volumes and the number of pulmonary metastasis (56, 57).

EMMPRIN is a multifunctional protein, which is moderately expressed on stroma cells, and overexpressed on many types of tumor cells. Among its many functions, EMMPRIN can induce the expression of VEGF and several types of MMPs. We have previously identified a specific short epitope as being responsible for the induction of VEGF and MMPs (65), and synthesized this epitope as an octa-branched MAP and vaccinated tumor-bearing mice with it (59). We show in three different implanted models and in two experimental metastasis models that the vaccination reduced angiogenesis by reducing MVD, VEGF, and MMP-9 concentrations. Additionally, the vaccination reduced tumor cell proliferation, increased macrophages and CTL infiltration into the tumor, and shifted the TME to allow more cytotoxicity toward the tumor cells, thereby reducing tumor size and the number of metastatic lung foci (59). In a DSS-induced colitis model that simulates the human autoimmune disease ulcerative colitis, we show that a similar effect occurs, where angiogenesis is reduced and infiltration of macrophages and CTLs to the colon tissue is increased, ultimately leading to improvement in the clinical score of the vaccinated mice relative to their controls (66).

### Clinical Studies

Currently, most clinical studies are at phase I or II, designed to test safety of peptide vaccination, toxicity, required dose, and induction of immune responses by the vaccine (immunogenicity), and not to estimate the efficacy of the vaccination. In some studies, a monotherapy approach was taken, vaccinating patients with single or multiple peptides, whereas in others a combination with chemotherapy was tested. These experiments are summarized in Table 2.

**Table 2:** Clinical trials for pro-angiogenic peptide vaccines.

**Antigen and type of peptide**	**Cancer type**	**Adjuvant used**	**Positive CTL response**	**Clinical response**	**Phase**	**HLA restriction**	**Combination**	**References**
**Monotherapy**								
VEGFR2-169 (9 aa)	Advance pancreatic cancer	Montanide ISA-51	11/18 (61%)	DCR 67%	I	A2402	Gemcitabine	(67)
VEGFR1-770 or VEGFR1-1084 (9 aa each):	Metastatic renal cell carcinoma	Montanide ISA-51	8/18 (83%)	SD (over 5 months)−8/18 (45%); PR−2/18 (11%)	I	A2402, A0201	None	(68)
**Multiple epitope vaccines**								
VEGFR1-1084 and VEGFR2-169 (9 aa each)	Advanced gliomas	Montanide ISA-51	7/8 (87/5%) to VEGFR1, 1/8 (12.5%) to VEGFR2	SD−25% (2/6) PD— 75% (6/8)	I	A2402	None	(69)
VEGFR1-1084 and VEGFR2-169 (9 aa each)	Advanced gastric cancer	Montanide ISA-51	18/22 (84%) to each of the peptides	PR−12/22 (55%) SD−10/22 (45%) DCR−100%	I/II	A2402	S-I and cisplatin	(70)
VEGFR1-1084, VEGFR2-169, RNF43-721, TOMM34-299, KOC1-508 (9–10 aa each)	Advances Coloreactal cancer (CRC)	Montanide ISA-51	18/18 (100%) to at least one of the peptides 10/18 (55%) to VEGFR1, 12/18 (66%) to VEGFR2	CR-1/18 (5.5%) SD-6/18 (33%) DCR 38.9%	I	A2402	None	(71)
VEGFR1-1084, VEGFR2-169, KIF20A-66 (9–10 aa each)	Resected pancreatic cancer	Montanide ISA-51	13/29 (44.8%) to VEGFR1 13/29 (44.8%) to VEGFR2	Median DFS of 15.8 months relative to 12 month of controls (only gemcitabine).	II	A2402	Gemcitabine	(72)
VEGFR1-1084, VEGFR2-169, KIF20A-66 (9–10 aa each)	Advanced pancreatic cancer	Montanide ISA-51	22/37 (59%) to VEGFR1 16/37 (43%) to VEGFR2	RR-12.1% PR-8/66 (12%) SD-41/66 (62%) DCR-74.2%	II	A2402	Gemcitabine	(73)
DEPDC1-294, URLC10-177, FoxM1-262, KIF20A-66, VEGERI-1084 (9–10 aa each)	Advanced gastric cancer	Montanide ISA-51	11/20 (55%) to VEGFR1	SD−10/22 (45%) PD−12/22 (55%)	II	A2402	None	(74)

*CR, complete response; SD, stable disease; PR, partial response; PD, progressive disease; DFS, disease-free survival; DCR, disease control rate (usually SD + PR)*.

Targeting VEGFR2, Miyazawa et al. (67) have vaccinated pancreatic cancer patients with a VEGFR2-derived peptide (VEGF-169, HLA-A2402 restricted) and vaccinated in combination with gemcitabine treatment, the standard care for pancreatic cancer patients with metastatic or recurrent disease. The vaccination was well-tolerated with no vascular adverse events (such as bleeding, thromboembolism, or hypertension) reported. Immune responses at the injection site were observed in 83% of the vaccinated patients, but only 61% of them exhibited stimulation of epitope-specific cytotoxic T cells, with reduced frequency of epitope-specific Tregs. Disease control rate (DCR) was 67%, including patients with stable disease (SD) or partial response (PR), whereas 33% exhibited progressed disease (PD).

Similar results were shown in the use of either the HLA-A0201-restricted peptide VEGFR1-770 or the HLA-A2402-restricted peptide VEGFR1-1084 for the treatment of metastatic renal cell cancer (RCC). Out of the 18 patients examined, 15 developed CTL responses specific to the injected peptide (83%), regardless of the dose injected. Two of the cohort exhibited PR, and five showed SD for over 5 months, so that the DCR was 55% (68).

To test the feasibility of peptide vaccination in high-grade glioma patients (including glioblastoma, anaplastic astrocytoma and anaplastic oligodendroglioma), eight patients were vaccinated with HLA-A2402-restricted peptides derived from the VEGF receptors VEGFR1-1084 and VEGFR2-169. Most patients developed positive immune responses to the VEGFR1 peptide (87.5%) and VEGFR2 peptide (12.5%), but this was not correlated to overall survival. However, a negative correlation that was found between plasma IL-8 levels and overall survival may suggest the use of IL-8 levels as a biomarker for vaccination efficacy (69). The authors suggest that targeting VEGF receptors may be more efficient than targeting VEGF alone, as these receptors can bind all VEGF family members, and may promote the killing of VEGFR-expressing tumor cells and endothelial cells.

Multiple epitopes (“cocktail”) vaccinations were tested in several studies. The pro-angiogenic VEGF receptors were targeted using a mixed “cocktail” vaccination that included the peptides VEGFR1-1084 and VEGFR2-169, with or without other antigens, and often in combination with a chemotherapeutic drug. No difference in the overall survival (OS) and progression free survival (PFS) was found if each peptide was injected in a different site or if all peptides were mixed together and injected in a single site (71). When patients were stratified between those that expressed the HLA-A2402 haplotype and those that did not, no significant change was observed (73, 74). The ability to generate a peptide-specific cellular immune response, which was tested by the IFNγ secretion of CD8^+^ T cells that were stimulated *ex vivo* with the peptide in ELISPOT assay, was correlated to disease free survival (DFS) rate or disease control rate (DCR) (72, 73), suggesting that the activation of an immune response was responsible for the clinical effect. In most studies, high percentage of the patients exhibited positive CTL responses to at least one of the vaccinating peptides. In one study, patients that had positive CTL responses to the VEGR2-169 peptides, but not those with immune responses to VEGFRI-1084 peptide, had significantly better prognosis (70). In contrast, another study showed better OS in patients that had positive CTL responses to VEGFR1-1084 but not to VEGFR2-169 (73). Thus, additional studies are needed to determine which receptor is the preferred target.

NRG-TNF is a drug consisting of the human TNFα protein fused to the CNGRCG peptide that targets it to aminopeptidase N (CD13), an enzyme overexpressed on newly formed tumor endothelial cells (75). NRG-TNF alters the vascular barrier and allows the increased uptake of chemotherapeutic drugs by the tumor cells, and improves immune cell infiltration. In a phase I/II clinical study, NRG-TNF was administered to patients with metastatic melanoma that were resistant to other drugs, together with one of the two peptides that were derived from melanoma-associated antigens, according to their HLA-A haplotype restriction. One peptide (NA17.A2) was derived from a spliced form of N-acetylglucosaminyltransferase expressed on 50% of melanoma patients, and another peptide (MAGE-3.A1) was derived from chain A of the MAGE 3 protein expressed on 70% of melanoma patients (76). All patients had increased serum levels of the chemokines MCP-1 and MIP-1β, suggesting inflammation and increased infiltration of immune cells into tumors. Additionally, immunohistochemistry in some lesions showed increased infiltration of macrophages (76). 6 out of 7 patients showed positive T cell responses to the peptides or to other melanoma antigens (due to antigen spreading) in the peripheral blood, and long-term survival (above 4 months) was demonstrated in 4 out of 8 patients (76). These results demonstrate the benefit of combination therapy that target the tumor vasculature and provides immunotherapy against tumor antigens.

In all the studies mentioned above, no adverse effects of grade 3 or higher were observed, and all doses examined were well-tolerated. However, limited rate of more severe adverse responses, especially neutropenia, were observed in some of the studies when peptide vaccination was combined with chemotherapy (70, 72, 73). The most common effects were erythema and pain at the site of injection. Thus, in accordance with other studies that targeted a wide range of non-angiogenic targets, peptide vaccination seems to be safe and well-tolerated. Of interest, some studies indicated that a better clinical outcome was generally observed in patients with a strong injection site responses (ISR), sometime reaching significance (72, 73).

## Summary and Conclusions

One of the problems in cancer immunotherapy is the set of defense mechanisms employed by the tumor to evade immune recognition, and especially its ability to alter antigens or lose their expression due to mutations. Especially pertinent to vaccination is the ability of tumors to reduce or lose the expression of HLA class I molecules, thereby avoiding efficient antigen presentation and immune response. As this makes targeting of tumor antigens more difficult, an alternative way might be to target antigens expressed on vascular ECs and induced in the tumor tissue by the angiogenic switch. This approach is effective even if these antigens are not expressed by the tumor cells, since ECs that stably express HLA molecules are the main targets of the vaccination, resulting in tumor cell suffocation and increased death due to reduced angiogenesis.

Using peptide vaccination is a promising approach to target angiogenesis. So far, targeting antigens by peptide vaccination in general, and attacking angiogenic targets in particular, have shown only limited therapeutic beneficial results, although most studies demonstrate stimulation of a peptide-specific immune response. However, all clinical studies exhibit safety and vaccines were well-tolerated with only mild adverse responses. Thus, once optimal conditions for vaccination are defined, peptide vaccination may be more advantageous than monoclonal antibodies that carry the risk of long-term ADA or rebound effect. These optimal conditions include target choice, peptide formulation, adjuvant and delivery systems, choice of patient populations that will better respond to treatment, and the vaccination regimen.

### Target Choice

Lessons learnt from cancer peptide-vaccinations that target a variety of TAAs suggest that targeting of TAAs exhibits only limited efficacy. This is explained by tolerance that retains only a limited T cell repertoire with low affinity to TAAs, by the ability of tumors to escape immune recognition by reducing or losing expression of MHC/HLA class I molecules, and by the immunosuppressive TME. Most clinical trials targeting pro-angiogenic proteins focused on VEGF and VEGFRs. However, these targets are problematic, as they can be compensated for by other members of their family or other pro-angiogenic proteins. One approach could be to use multiple-epitope vaccines that would include VEGF, VEGFR1, VEGFR2, FGF-2, and additional pro-angiogenic targets injected together as a cocktail. Another approach would be to identify additional pro-angiogenic protein targets. Such proteins could affect ECM remodeling (e.g., heparanase), or be overexpressed on the tumor vasculature and/or tumor cells (e.g., EMMPRIN). Thus, since EMMPRIN is expressed on tumor cells, leukocytes (especially Tregs) and tumor vasculature, targeting it could directly and simultaneously attack tumor cells, disrupt tumor vascularization, and alleviate immune suppression. The ideal target would be a protein that is essential to tumor growth and dissemination, so that the tumor cannot afford to reduce its expression. Preferably, such a target would be expressed on both tumor cells and tumor vasculature.

### Peptide Formulation

The vast majority of peptide vaccines tested so far, including those targeting angiogenic proteins, are based on short peptides, and only few studies used SLPs to target pro-angiogenic proteins. All of these studies, for the most part, did not yield tumor regression in pre-clinical studies or complete response in clinical studies. T cells that expand after vaccination *a priori* have only low affinity and avidity to tumor antigens, due to elimination of high affinity T cells by central and peripheral tolerance, and so are not sufficient to drive strong anti-tumor responses. Better results may be obtained by using modified peptides, especially the multiple antigenic peptide (MAP) modification. Modification of the peptide seems to be a crucial strategy to elicit a sufficiently strong immune response. Therefore, it is highly recommended to introduce modifications to the peptide formulation in future experiments. Future research should attempt to identify the best type of modification that would elicit a strong immune response against the modified peptide, thus overcoming tolerance, yet allowing cross-reactivity with the native antigen.

### Adjuvant and Delivery Systems

Most studies used IFA or Montanide ISA-51 and only recently other compositions that include TLR ligands or GM-CSF are being evaluated. It seems that the choice of adjuvant may be critical in light of evidence demonstrating entrapment of T cells in the vaccination site, and it is still not fully understood whether this occurs only for short peptide vaccines or may also occur using SLPs or modified peptides. Therefore, much work should be devoted to identifying the optimal adjuvant for cancer peptide vaccines.

### Patient Populations

Selection of patients for clinical studies is usually biased, limiting our possibility to evaluate vaccine efficiency. Patients that participate in clinical studies are often terminally ill, far-advanced patients with high grade and stage tumors and/or widespread metastases that have already shown refractoriness to treatments with chemotherapy or radiotherapy, and whose immune system is already compromised. Therefore, the window of opportunity to vaccinate efficiently is long-passed in these patients. It is conceivable that patients with early stage disease could potentially benefit more from peptide vaccination. Studies should look at the efficacy of vaccination in sub-populations according to the stage of the disease.

### Vaccination Regimen

Usually peptide vaccination is performed as a standalone approach or a<underline >monotherapy, yielding only poor clinical benefits, and when combined with chemotherapy, improvement is noticeable. Therefore, future investigations should identify the best modality of treatment to combine with peptide vaccination, which would yield significant clinical improvement. Since peptide vaccination is about triggering the immune system and restoring its anti-tumoral effects, it is logical to examine a possible combination between peptide vaccination and checkpoint inhibitors, a combination likely to repolarize the immune system toward the desired effect. Experiments with monoclonal antibodies revealed that anti-VEGF inhibits the expression of checkpoint inhibitors such as PD-1, CTLA-4, LAG-3, and TIM-3, preventing the exhaustion of CD8^+^ T cells, and suggesting a mechanism that could explain a synergistic effect of anti-PD-1 and anti-VEGF (77). In view of the recent success in combining checkpoint inhibitors with other anti-angiogenic treatment modalities (78), this combination approach might also be highly effective for anti-angiogenic peptide vaccines and should be explored further. Although data are lacking at the moment, it will be interesting to see future developments using a combination of neoantigen-derived peptides with peptides targeting the tumor vasculature, and to explore whether such combinations enhance the anti-tumoral response and increase clinical success.

Of note, pathological angiogenesis that results from an imbalance between pro- and anti-angiogenic factors is associated with many types of chronic inflammatory diseases. While cancer diseases are one form of chronic inflammation, angiogenesis is also essential to the progression of autoimmune and inflammatory diseases (79, 80). However, with the exception of our previously mentioned study on a DSS-induced colitis model (66), other studies on peptide vaccination targeting angiogenic proteins in autoimmune disease models were not found. Therefore, examining the potential of targeting angiogenesis in such conditions is strongly indicated.

In conclusion, the fact that most peptide vaccinations demonstrated poor clinical benefits is the main difficulty facing the development of new peptide vaccines. On the other hand, peptide vaccination is safe and well-tolerated, suggesting clear potential advantages over other modalities of treatment. The data presented here suggests that peptide vaccination, especially against angiogenic targets, is still a viable option, if peptides are modified, targets are well-selected and an optimal adjuvant is used. Additional possibilities of using peptide vaccines as adjuvant therapy to other treatment modalities still await more exploration. Still, targeting angiogenic proteins may be a double-edged sword, as these proteins may be physiologically expressed in normal tissues as well. In this case, stimulating the immune system against these proteins could risk triggering autoimmunity and cause catastrophic results. However, so far, peptide vaccination in general, and that of pro-angiogenic targets in particular, has been well-tolerated and showed no adverse responses, suggesting that the immune system is directed in a selective manner to the tumor site. The mechanisms that might explain such a phenomenon should be intensively studied. Once such mechanisms are better understood, they could be manipulated at need to avoid autoimmune diseases and promote the use of peptide vaccination for the treatment of cancer diseases.

## Author Contributions

MR collected the data, organized, drafted, and wrote the paper.

### Conflict of Interest Statement

MR is an inventor of a patent (US Grant US9688732B2, EP application EP2833900A4) related to peptide vaccination approach described in the manuscript.
